# The conceptualisation of health and disease in veterinary medicine

**DOI:** 10.1186/1751-0147-47-71

**Published:** 2006-11-07

**Authors:** Stefan Gunnarsson

**Affiliations:** 1 Section of Animal Hygiene, Department of Animal Environment and Health, Swedish University of Agricultural Sciences (SLU), P.O. Box 234, S-532 23 Skara, Sweden

## Abstract

**Background:**

The concept of health, as well as the concept of disease, is central in veterinary medicine. However, the definitions "health" and "disease" are not generally acknowledged by veterinarians. The aim of this study was to examine how the concepts "health" and "disease" are defined in veterinary textbooks.

**Methods:**

Veterinary textbooks in several disciplines were investigated, but only textbooks with explicit definitions of the concepts were selected for examination.

**Results:**

Eighty out of the 500 relevant books within veterinary medicine were written for non-veterinarians. Eight percent of the books had an explicit definition of health and/or disease. More frequently, textbooks written for non veterinarians did have definitions of health or disease, compared to textbooks written for professionals. A division of health definitions in five different categories was suggested, namely:

1. Health as normality, 2. Health as biological function, 3. Health as homeostasis, 4. Health as physical and psychological well-being and 5. Health as productivity including reproduction.

**Conclusion:**

Few veterinary textbooks had any health or disease definition at all. Furthermore, explicit definitions of health stated by the authors seemed to have little impact on how health and disease are handled within the profession. Veterinary medicine would probably gain from theoretical discussions about health and disease.

## Background

The concept of health, as well as the concept of disease, must be regarded as essential to veterinary medicine. Nevertheless, it appears to be uncommon that broader discussions about these basic concepts occur within the veterinary society. The increasing diagnostic possibilities to identify diseases make it crucial to define disease and health, as this basic distinction gives the very fundament of disease classification.

The naive definition of health in veterinary medicine seems to be that health is no more than the very absence of disease, which can be considered as a dichotomous definition. This position is often the case for basic assumptions in e.g. epidemiology, where the disease frequency commonly is calculated based on that disease is binary, which means that either the animal has the disease or it does not have the disease.

The epidemiological methods used to investigate the excellence of a disease test, e.g. the sensitivity and the specificity of a serological method, is built on the understated assumptions of well-defined concepts of disease and health. However, it seems to be rare that any evaluation of diagnostic methods is based on any scrutiny or more precise definitions. For example in a well known textbook in veterinary epidemiology quite extended parts of the book emphasize the theoretical problems associated with diagnostic tests [1]. But the concepts of health and disease are just briefly reviewed and Martin and co-authors refer that productivity is commonly used as a surrogate definition of health in veterinary medicine, although this has been seriously questioned by several others [e.g. [2]].

Pathology has always been an essential part of veterinary medicine. It is more or less self-evident that the pathologist should be able to determine health from disease in order to put an accurate diagnosis. Nevertheless, it seems rare to find any explicit definition of these central concepts or even to find the subject being investigated further.

No matter in what area the veterinarian is active, the daily work involves, directly or indirectly, health and disease in animals. Therefore, it is logical to examine how different concepts of disease are used within veterinary medicine, and to examine textbooks in veterinary medicine, e.g. veterinary pathology, internal medicine and epidemiology.

The aim of the present study is to examine how the concepts of "health" and "disease" are defined in veterinary textbooks in pathology, epidemiology, internal medicine and other areas. Definitions of health and disease found in various textbooks of veterinary medicine are examined and categorised. Furthermore, the implications of the different health definitions are discussed.

## Methods

The veterinary textbooks at the libraries of the Swedish University of Agricultural Sciences (SLU) were examined during the spring of 2003. The literature investigated compiled veterinary textbooks in several disciplines, such as pathology, internal medicine, bacteriology and immunology.

The approach of examining the literature was to perform a scanning of the tables of content, indexes and introductory chapters of every volume. The scanning was done to identify any definition or theoretical discussion about health or disease. Only textbooks with explicit definitions of health and disease were selected for further examination. Thus, this study does not investigate implicit definitions of health and disease, as the identification of such definitions would demand far more advanced analyses, as well as, there would be an increased risk of getting arbitrary definitions.

## Results

About 80 out of the 500 relevant books I found within veterinary medicine were written for non-veterinarians, such as veterinary nurses, farmers or the common man. Thirty-nine of the 500 books (8%) comprised any explicit form of, more or less developed, definition of health and/or disease. Twenty-two books out of these 39 were written for veterinarians or veterinary students, five were veterinary dictionaries, two were handbooks for veterinary nurses and ten were written for farmers or animal owners. Twenty-five volumes out of the 39 were written in English, four were in German, five were in Swedish, two were in Norwegian, two were in Danish and one volume were written in French.

Four out of 39 volumes were available in several editions. These different editions were counted as single handbooks, and special attention was paid to any added or extended health or disease definition. Different editions revealed an extended or modified definition of health [3-7]. Other handbooks had unchanged text sections about health/disease throughout several editions, e.g. a German textbook for veterinary nurses [8-10].

A higher proportion of textbooks written for non-veterinarians contained definitions of health or disease, compared to textbooks written for veterinarians (15% versus 7%). The three textbooks about alternative veterinary medicine with explicit health definition were all written for laymen and specifically covered homeopathic treatment of animals [11-13]. These books were not only giving a definition of health from the point of view of alternative veterinary medicine, but also a health definition of what the authors saw as conventional medicine, which they rejected. However, there were no references to other publications in these books.

Most books had only one single definition of health or disease. The exception was, apart from the books in alternative medicine, a dictionary of veterinary medicine where alternative definitions of health were given [14,15].

## Discussion

Few veterinary textbooks had any health definition at all. In general, common textbooks of pathology were lacking any suggestion of how to define health and disease. Typically, the textbooks of pathology and internal medicine comprised chapters about each system of organs, i.e. one chapter about diseases of the integument, one about disease of the respiratory system, one about disease of the digestive system and so on. These textbooks contained contributions from several authors, responsible for text sections of the diseases of an organ system or a group of diseases. Then the book chapters were put together by the editors [e.g. [16,17]].

### Categories of health definitions

A subdivision of health definitions is in itself arbitrary. However, such a division may increase the possibility to comprehend heterogeneous items of more or less developed definitions of health and disease. The subdivision presented here should be regarded as a tool to bring order in this study, rather than to establish a definition of schools of the subject once and for all.

Different approaches to the concept of health and disease were found in the literature reviewed and they can be split into the following five categories:

1. Health as normality

2. Health as biological function

3. Health as homeostasis

4. Health as physical and psychological well-being

5. Health as productivity including reproduction

#### 1 Health as normality

It seems common to define health and disease in terms of normality or abnormality. However, definitions referring to health as normality were usually very limited in their extension and sometimes only fragments were found. An example of normality definition comes from a manual for animal health auxiliary personnel written by the Food and Agriculture Organization of the United Nations [18], where a healthy animal is described to have a normal appearance and behaviour. The animal should have normal features, including normal body position and movements. This definition is actually not a definition of the concept of health as such, but an operational definition of clinical health, assuming normality.

John Webster [19] has a similar approach to health in his disease definition which relates disease to a normal state and that "ill health always appear as departure from normality". Webster also puts our attention to the importance of getting clinical experience of the normal health animal in order to understand the normal variation of healthy animals.

In a German veterinary dictionary 'disease' is defined as a disturbance of the normal function of the body [20]. A similar definition is given by Arnall and Keymer [21], who define health as a soundness of the body and an absence of disease, which assume that there is a normality of health. However, the authors are referring to diverse definitions of health without giving a clear opinion of their own.

Baker and Greer [22] give a semantic analysis of the disease concept before they propose a normality-based health concept. They define disease "as 'not at ease' because the prefix 'dis' denotes reversal or separation from the root 'ease'. Animal ill health is synonymous with the word 'disease'."

In the previous definitions there are clearly stated attempts to base the health concept on normality, but elements of biological function can also be introduced as relevant to normality definition [22,23]. Particularly Baker and Greer [22], in a passage about that "structural defect or functional impairment of the animal body", implies that they do not only define health as normality, but that they also think that health is linked to a biological functioning.

#### 2 Health as biological function

Slauson and Cooper [24] propose in their textbook about comparative pathology that disease can be seen as a manifestation of malfunctioning physiology or physiology that has gone wrong. They think that disease ultimately reflects structural or functional alteration in the cells of which all living things are made, which is the common idea within pathology.

Gillespie and Timoney [25] give a similar definition of disease, namely that a disease is defined disturbances of proper performance of body functions, or as they put it: "Disease may be defined as an alteration of the state of the body, or of some of its organs, which interrupts or disrupts the proper performance of the bodily functions. Functional disturbance soon is manifested by physical signs which usually can be detected by others."

The pathologist Norman F. Cheville [26] expresses that veterinary pathology is abnormal biology in a wide sense, and that "pathology is essentially the search for and the study of lesions, the abnormal structural and functional changes that occur in the body."

The idea of health as based on biological functioning is related to the idea of homeostasis, which could be said to develop the idea of functioning more precise by setting the boundaries of functioning.

#### 3 Health as homeostasis

Health defined as homeostasis is an old idea, but still it is a common way of looking at health and disease. The concept of homeostasis is related to the maintenance of a delicate balance within the organism or within the processes in the organism. Here is one example: "Disease arises when the normal interplay of the body functions is disturbed. It is depending on the constitution of the organism, on the disposition for disease and on the disease causes that influence the body, e.g. infectious agents, environmental factors, toxic substances and alteration of nutrients" [[8-10], translated from German by the author].

The idea of homeostasis seems so firmly established within the discipline of physiology that it is not just regarded as an approach to describe physiological processes; it is considered to be a law of nature in the same way as the idea of evolution is viewed as a law of nature not a biological theory.

The idea of health as homeostasis is commonly used within veterinary homeopathy. In the three homeopathy books found, the discussions about health were quite extended and the homeostasis idea was contrasted to the assumed ideas of conventional medicine. The homeostasis idea was expressed as being holistic, which can be illustrated by Christopher Day [12], who writes about homeopathic veterinary medicine in comparison to conventional medicine, as he looking at it.

"In holistic terms, the word 'health' implies the concept of a mind and body together in harmony with the environment. When the organism, comprising the mind and the body, is out of the harmony within itself or with its environment, then we have the state of disease (literally dis-ease).

Modern conventional medicine tends to view disease as a set of signs and symptoms, recognisable combinations of which are called 'disease'. Each of these given a name and is assumed to have an identity of its own. In holistic medicine we view disease differently. We see the signs or symptoms simply as a result of, and expression of the body's reaction to, the disease forces which impinge upon it, threatening to disturb its internal equilibrium. Like all systems in equilibrium, the body – a very sensitive and active equilibrium system-reacts to disturbing forces in an attempt to retain or regain balance." [12]

Homeopathic health definitions are often stated to be "holistic" and based on the idea that health is depending on homeostasis. However, as health definition found in conventional veterinary textbooks also can be based on the homeostasis idea, it can not be deduced that homeostatic thinking logically leads to homeopathic medicine. One example of this is that Lagerlöf, Hallgren and Ekesbo [27] define health as a state where all organs are in a delicate balance within the organism and with the surrounding world. This definition has been further refined by Ekesbo [27,28]. The authors Lagerlöf, Hallgren and Ekesbo clearly belong to classic veterinary medicine and not the homeopathic school.

#### 4 Health as physical and psychological well-being

It is common in the debate about animal welfare to propose definitions of health that includes psychological aspects of health [2,29,30]. However, it seems uncommon that welfare and well-being are included in the health definitions in veterinary textbooks. One exception is Martin and co-authors [1] that start off with giving references to the human health definition stated by the World Health organisation (WHO), i.e. that health is "a state of complete physical, mental, and spiritual well being", but they also think that productivity is a substantial part of health in farm animals.

If health is defined as physical and psychological well-being, there are problems associated with applying the definition to all animals. Domestic animals are more or less subordinate to human interests and this is most obvious regarding farm animals. A health definition that puts priority to the physical and psychological well-being of an animal is misleading in relation to the general purpose of livestock production.

In their veterinary dictionary, Blood and Studdert [14,15] express similar thoughts as Martin and co-workers [1], as they claim that health is "a state of physical and psychological well-being and of productivity including reproduction". Although this definition is very short, it mixes two different approaches to health, which could be regarded as contradictory. At first there may not be any conflict between well-being and productivity, but how do we make priority between these two aspects if they are in conflict. Are both parts necessary to fulfil in order to be healthy? This dual health definition leads us to the last category of health definitions, which includes productivity aspects.

#### 5 Health as productivity including reproduction

The previous definitions could be seen as universal, i.e. the health definitions could be applied to all animals including humans. However, it would probably be hard to use a health definition that equalises health to productivity, as a general health definition for humans or other non-producing animals, e.g. pets.

The idea that health in animals solely is the same as productivity is quite uncommon in the literature, although it is e.g. proposed by C. S. G. Grunsell in Black's veterinary dictionary [31], where it is stated that health "is now more accurately regarded as a state of maximum economic production".

Even if it is rare to define health purely in productivity terms, several authors incorporate elements of reproduction and productivity in their definition of health for farm animals. For example, Aspinall [32] states that "a healthy animal grows, reproduces, and behaves in a manner which has come to be regarded as normal for its species and type".

In the book "Veterinary medicine: A textbook of the diseases of cattle, sheep, pigs, goats & horses" [available in several editions e.g. [7]], it is proposed that disease traditionally has been defined as "abnormality of structure or function" and that it has long been widened to embrace subclinical diseases. Furthermore, it is reported that the concept of disease also includes "failure to produce" at expected level of nutritional supply and environmental quality. But the authors also declare that "the detection of residues of disqualifying chemical in foods of animal origin will also come to be included within the scope of disease". This extended definition of health is not just relevant to the individual animal but also cover the environmental effects.

David Sainsbury [33] proposes a slightly modified health definition related to productivity. He introduces the term "positive health", which he defines as" the provision of a complete diet, an environment that is optimal for the animal's physiological needs, comfortable to the animal's senses, in which the animal is secure and free from fear, and with no undue challenge by pathogenic micro-organisms or predators". Sainsbury's definition is interesting as it is not just a describing health, but the definition is also normative, i.e. telling us how health should be. He claims that "good health is the birthright of every animal that we rear, whether intensively or otherwise". He is also extending health beyond the subjective health state of the animal, as he argues that medication to control disease is not giving true health to the animal although the animal appears to be healthy.

### General discussion

It was rare that veterinary textbooks gave explicit definitions of health and disease. The main reason for that may be that the general purpose of the textbooks was not to investigate what is healthy or what is diseased in principle, but to describe diseases and their causes and consequences.

It was common that textbooks use different aspects of health definition [20]. This makes it harder to actually know what the main health idea is in a textbook. When an explicit definition of health was presented in a textbook it was almost always given without references to other health definitions. It can be assumed that the reason for this is that the authors were focused on the general topic of the book and not philosophy of health.

The general debate of welfare in animals was never referred in veterinary textbooks within this study, and the authors seemed to write in isolation from other authors' definition of health and disease. However, there were exceptions from this in the books of veterinary homeopathy. All three books about veterinary homeopathy found were referring to what the authors found as incorrect health definitions, i.e. the mechanistic approach they attributed the school of classic veterinary medicine [11-13].

Homeopathic health definitions were stated to be 'holistic' and were mainly based on the idea that health is depending on homeostasis. However, as health definition of conventional veterinary textbooks also could be based on the homeostasis idea, it can not be concluded that 'homeostasis thinking' logically leads to homeopathic medicine, as mention above. This observation may illustrate that the basic theory behind a health definition do not have inevitable consequences for the veterinary practice. It seems like the explicit definition of health stated by an author had little or no impact on how health and disease were handled within the profession.

Veterinary medicine would probably gain on a more intense discussion about the concept of health. A veterinarian that never contemplates the health concept would merely be an "animal mechanic", who just needs to find out in what way the animal is broken, and then fix it according to the manual. If the concepts of health and disease were more clearly defined, the communication of e.g. zoonotic hazards to the public would become more robust. Furthermore, the general discussion of animal welfare issues would become more relevant if the basic concepts of health and disease were more commonly penetrated in the debate.

## Conclusion

The concepts of health and disease are rarely given explicit definitions in veterinary textbooks. The definitions found could be split into five categorised of health definitions. There was no obvious connection between stated health definition and medical approach, i.e. classic veterinary medicine or alternative veterinary medicine. Veterinary medicine would gain from a broader theoretical discussion about health and disease.

## Competing interests

The author(s) declare that they have no competing interest.
